# Arthroscopic cartilage regeneration facilitating procedure for osteoarthritic knee

**DOI:** 10.1186/1471-2474-13-226

**Published:** 2012-11-21

**Authors:** Shaw-Ruey Lyu, Chia-Chen Hsu, Chih-Wen Lin

**Affiliations:** 1 Joint Center, Buddhist Dalin Tzu Chi General Hospital, 2, Min-Shen Road, Chiayi, 62247, Taiwan; 2 Department of Medical Imaging, Buddhist Dalin Tzu Chi General Hospital, Chiayi, Taiwan; 3 Tzu Chi University, Hualien, Taiwan

**Keywords:** Osteoarthritis, Knee, Medial plica, Arthroscopy, Cartilage, Regeneration

## Abstract

**Background:**

The effectiveness of arthroscopic treatment for osteoarthritic knee is a controversy. This study presents the technique of a novel concept of arthroscopic procedure and investigates its clinical outcome.

**Method:**

An arthroscopic procedure targeted on elimination of focal abrasion phenomenon and regaining soft tissue balance around patello-femoral joint was applied to treat osteoarthritis knees. Five hundred and seventy-one knees of 367 patients with osteoarthritis received this procedure. There were 70 (19%) male and 297 (81%) female and the mean age was 60 years (SD 10). The Knee Society score (KSS) and the knee injury and osteoarthritis outcome score (KOOS) were used for subjective outcome study. The roentgenographic changes of femoral-tibial angle and joint space width were evaluated for objective outcomes. The mean follow-up period was 38 months (SD 3).

**Results:**

There were 505 knees in 326 patients available with more than 3 years follow-up and the mean follow-up period was 38 months (SD 3). The subjective satisfactory rate for the whole series was 85.5%. For 134 knees with comprehensive follow-up evaluation, the KSS and all subscales of the KOOS improved statistically. The femoral-tibial angle improved from 1.57 degrees (SD 3.92) to 1.93 degrees (SD 4.12) (mean difference: 0.35, SD 0.17). The joint space width increased from 2.02 millimeters (SD 1.24) to 2.17 millimeters (SD 1.17) (mean difference: 0.13, SD 0.05). The degeneration process of the medial compartment was found being reversed in 82.1% of these knees by radiographic evaluation.

**Conclusions:**

Based on these observations arthroscopic cartilage regeneration facilitating procedure is an effective treatment for osteoarthritis of the knee joint and can be expected to satisfy the majority of patients and reverse the degenerative process of their knees.

## Background

Osteoarthritis (OA) of the knee affects a large population worldwide and is associated with an extremely high economic burden largely attributable to the effects of disability, comorbid disease, and the expense of treatment [1]. To date the established therapies have been directed towards symptom control. Since the initiating events that result in the cartilage degradation are poorly understood, there has been very limited success in demonstrating disease modification in clinical trials of potential therapies [2].

Arthroscopy for the management of OA knees, as a relative minor procedure, is popular in spite of the controversy concerning its effectiveness. Joint lavage [3,4], debridement [5-7], abrasion arthroplasty [7-11], and microfracture [12-14] are among the most commonly employed arthroscopic procedures for OA knees. The precise mechanism and consensus by which these procedures improve the course of degenerative conditions of the knee have not been established. A recent retrospective review of the current literature on the arthroscopic treatment of OA knee [15] has demonstrated limited evidence-based research to support the use of arthroscopy as a treatment method for OA knee.

In 2008, a concept of arthroscopic medial release (AMR) for the treatment of osteoarthritis of the medial compartment of the knee joint was reported [16]. The clinical outcome of this series lured us to believe that, by eradication of the abrasion phenomenon between the tight, fibrotic and hypertrophied medial structure and the adjacent medial femoral condyle, the pain of most patients could be reduced and the degenerative process in the medial compartment of these patients might be decelerated or arrested. In this report, we propose a concept of arthroscopic cartilage regeneration facilitating procedure (ACRFP) that combines arthroscopic medial release (AMR), percutaneous lateral release (PLR) and conventional debridement as a rationale for the deliberate arthroscopic management of OA knee. We hypothesized that the elimination of the detrimental factors including synovitis, chondral flaps, and abnormal focal stress caused by medial abrasion and lateral compression phenomena will provide a preferable environment for the regeneration of damaged cartilage. The details of this procedure and its clinical results are reported in this study.

## Methods

### The patients

In a one-year duration, 571 knees of 367 patients with different grades of OA received this procedure. There were 78 (19%) male and 330 (81%) female. The age ranged from 29 to 82. The mean age of these patients at the time of surgery was 60 (SD 10). There were 406 knees (80.4%) of 269 patients received AMR and 99 knees (19.6%) of 57 patients received AMR+PLR. The anteroposterior standing and Merchant’s axial radiographs were evaluated for the severity of degeneration for each compartment. The radiographic grading of each compartment according to Kellgren and Lawrence [17] was given by the senior author who was the single surgeon performed all of the procedures. The inclusion criteria were patients with OA knee of any grade involving medial compartment or combined with patellofemoral compartment and have been treated conservatively including non-steroidal anti-inflammatory drugs, nutrition supplements (glucosamine sulfate and/or hyaluronic acid injection) and physiotherapy for more than six months. The exclusion criteria were apparent lateral compartment OA (radiographic grading of III or IV or arthroscopic grading of III or IV by modified Outerbridge classification [18]), instability due to previous ligament injury, major meniscus tear, or OA due to trauma. This study was approved by the Research Ethics Committee of Buddhist Dalin Tzu-Chi General Hospital, which has been certificated by the Department of Health, Taiwan (IRB Approval Number: B09704022). Informed consents were obtained from all patients participating in the study.

### Radiographic Protocol

The standing extended anteroposterior radiographs of the knee were obtained at 55 KV with a focus of 1.25 mm^2^ and a focus-to-film distance of 100 cm, using 35 × 43 cm computed radiographic film (Kodak Digital Science, CR 400). Radiographs of the extended knee were obtained with the patella in central position [19]. To assess the patellofemoral joint space, Merchant’s view was obtained at 45 degrees of knee flexion with X-ray beam projected caudad at 30 degrees. For magnification correction in each examination, a 4.0-mm steel ball was affixed to the skin over the head of the fibula (standing extended AP view) and center of patella (Merchant’s view) to permit correlation of the joint space width measurements [20].

### Surgical procedure

All procedures were performed under spinal or general anesthesia. Bloodless surgical field was obtained by pneumatic tourniquet. A 4 milimeters 30 degrees arthroscope with 6.5 milimeters sheath was used. An electronic shaver and hand instruments were used as necessitated.

#### Arthroscopic examination

**Figure 1:**
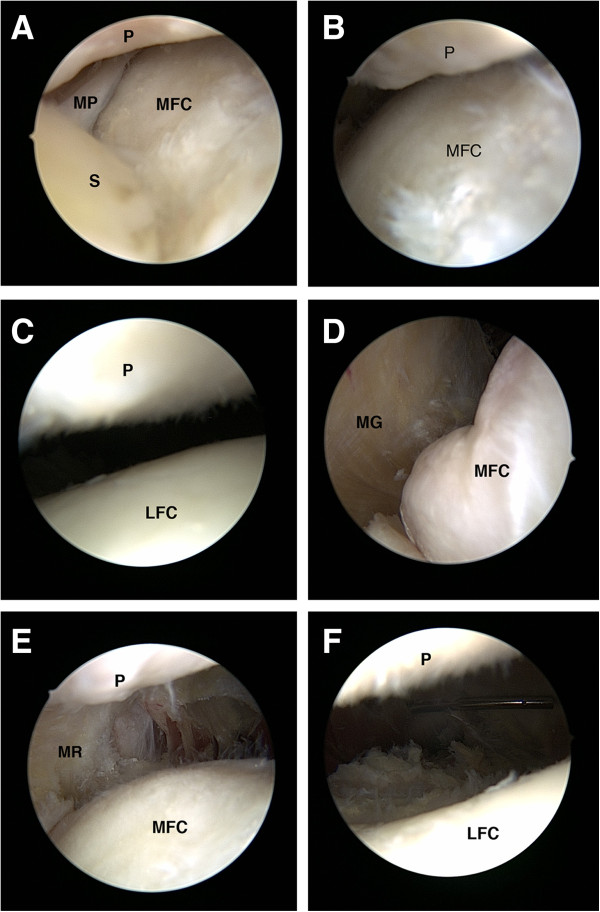
**Different stages of ACRFP.A**, typical presentation of medial abrasion phenomenon, the space between patella (P) and medial femoral condyle (MFC) was occupied by fibrotic media plica (MP) and inflammatory synovium (S); **B**, before medial release, the space between patella (P) and medial femoral condyle (MFC) was tight; **C**, before lateral release, the space between patella (P) and lateral femoral condyle (LFC) was tight; **D**, after eradication of medial abrasion syndrome, medial gutter (MG) could be clearly visualized; **E**, after medial release, the medial facet of the patellofemoral joint was opened and the medial retinaculum (MR) could be visualized; **F**, after lateral release, the space between patella (P) and lateral femoral condyle (LFC) was opened.

Through inferolateral portal, routine arthroscopic examination was performed. Special attention was given to the presentation of medial plica related medial abrasion phenomenon or focal synovitis over the inferomedial region of the patella (Figure 1A). The tightness and balance of patellofemoral joint was then evaluated (Figure 1B, C). If the medial or lateral facet could not be easily passed by the scope, it was recognized as over-tightness. Patients having apparent lateral compartment OA (grade III or IV by modified Outerbridge classification), major meniscus tear which needs partial meniscectomy or instability due to ligament injury were excluded from this study.

#### Medial release

For the knees with medial abrasion phenomenon (Figure 1A), medial release [16] (Additional file 1: Video 1) was performed to eradicate the inflammatory tissue occupying the space over the inferomedial region of the patella including ligamentum mucosum, fibrotic or inflamed synovium, capsule and distal part of the medial plica. The tight and obliterated medial facet of patellofemoral joint (Figure 1B) was then released by resection of the fibrotic synovium, capsule and proximal part of the medial plica (Figure 1D). Sometimes, medial retinaculum including fibrotic fascia of pes anserinus should be released. The adequacy of the medial release was checked by passing the tip of the scope under the patella and verified if the previously tightly closed medial patellofemoral joint space could be easily opened and the medial retinaculum visualized when the knee was put in full extension position (Figure 1E).

#### Percutaneous lateral release

For the knees with lateral compression syndrome (Figure 1C), lateral release was performed by inserting the No.11 scalpel into the inferolateral portal and cut the lateral retinaculum percutaneously. The extent and adequacy of the release could be evaluated by direct vision through arthroscope (Figure 1F).

#### Synovectomy and chondroplasty

Any focal synovitis or loose chondral flaps on the cartilaginous surface was removed as conventional arthroscopic debridement for osteoarthritis of the knee. At the end of the procedure, thorough irrigation was performed to remove any debris in the knee joint. No bony procedure such as drilling or microfracture was performed.

### Post-operative management

Suction drain was used for 24~48 hours. The involved limb was protected by elastic bandage for one week. Full range of motion, full weight bearing and free ambulation were allowed as tolerated. The patient was discharged 2 days after the operation. Home exercise programs, including active range of motion and quadriceps strengthening, were emphasized. No supplementary treatment including oral glucosamine sulfate, steoid injection or intraarticular injection of hyaluronic acid was given during the whole post-operative follow-up period.

### Follow-up and evaluation of clinical outcome

The patients were evaluated monthly for three months. Thereafter, they came back yearly for outcome assessment. The comparisons of both pre- and post-operative Knee Society score (KSS) and knee injury and osteoarthritis outcome score (KOOS) were used for outcome evaluation. Subjective satisfaction was assessed by direct question using a categorical scale prepared for this study: excellent, free of symptoms, no limitation in activities; good, greatly improved, occasional pain, normal activities; fair, same as pre-operative condition, no improvement; and poor, has received or considered further operative treatment. The outcome was regarded as satisfactory if subjective satisfaction was rated as “excellent” or “good”. The inquiry into subjective satisfaction and the evaluation of KSS and KOOS were conducted by nursing specialists. All investigations focused on individual knees in bilateral cases.

### Evaluation of x-ray outcome

The radiographic outcome was interpreted by comparing the pre-operative standing extended anteroposterior and Merchant’s views with the ones taken at the last visit by digitized assessment (Image-Pro, Media Cybernetics, Inc., Bethesda, MD, USA). The interpreting parameters include: femoral-tibial angle (FTA), minimum joint space width (MJSW) [21], lateral tilt angle of patella [22], and the surface contour of the joint lines. Simplified outcomes of better, the same or worse was given to each knee after evaluation of the pre- and post-operative radiographs. The anatomic axis (femoral-tibial angle) was defined as the angle formed by the intersection of 2 lines originating from points bisecting the femur and tibia and converging at the center of the tibial spine tips [23]. The same radiologist performed these measurements and interpretations.

### Statistical analysis

All values were presented with means and standard deviations. Comparisons were made using one-way analysis of variance (ANOVA) to detect differences in the distribution of patient age in each grade of osteoarthritis. Statistical analysis for comparing preoperative and postoperative KSS, KOOS, femoral-tibial angle, and joint space width was performed using the paired *t* test. *P* < 0.05 was considered to be statistically significant. All statistical analysis was carried out using JMP, the Statistical Discovery Software (Version 5.0.1.2, SAS Institute Inc., Cary, NC, USA).

## Results

There were 505 knees in 326 patients available with more than 3 years follow-up and the mean follow-up period was 38 months (SD 3). Ten of 59 knees (16.9%) with grade I osteoarthritic knees in six patients; twenty-five of 235 knees (10.6%) with grade II osteoarthritis in fifteen patients, twenty-six of 233 knees (11.2%) with grade III osteoarthritis in seventeen patients, and five of 44 knees (11.4%) with grade IV osteoarthritis in three patients were lost to follow-up. The total follow-up rate was 88.5%. One hundred and thirty-four knees returned and completed thorough outcome study including x-ray examination, KSS and KOOS evaluation. Another 371 knees completed the subjective outcome evaluation by telephone questionnaires. All of the knees were found to have the structure of pathologic medial plica. The mean age of these patients at the time of surgery was 60 (SD 10). The mean age and distribution of different grade of OA stratified by the procedure they received were shown in Table 1. There were 406 knees (80.4%) of 269 patients received AMR and 99 knees (19.6%) of 57 patients received AMR+PLR. The mean age of the patients were positively correlated with the severity of OA by ANOVA in both surgical groups. In the group received AMR+PLR, the F/M ratios were more prominent compared to that of the AMR group.

**Table 1:** Age and sex distribution of different grade of medial compartment OA stratified by type of surgery

	**AMR**		**AMR + PLR**		**Total**	
Stage	Age (SD)/No.	F/M (Ratio)	Age (SD)/No.	F/M (Ratio)	Age (SD)/No.	F/M (Ratio)
I	52 (9)/37	30/7 (4.3)	55 (10)/12	11/1 (11)*	53 (9)/49	41/8 (5.1)
II	58 (11)/163	123/40 (3.1)	59 (11)/47	43/4 (10.8)*	58 (11)/210	166/44 (3.8)
III	64 (8)/170	147/23 (6.4)	63 (5)/37	32/5 (6.4)	64 (7)/207	179/28 (4.6)
IV	62 (7)/36	30/6 (5.0)	66 (1)/3	3/0 (NA)*	62 (7)/39	33/6 (5.5)
Total	60 (9)/406	330/76 (4.3)	64 (11)/99	89/10 (8.9)*	60 (10)/505	419/86 (4.9)
	p<0.0001, R^2^=0.15		p=0.019, R^2^=0.07		p<0.0001, R^2^=0.13	

* Statistically significant compared to that of the AMR group by comparisons for each pair using paired *t* test (p<0.05).

### Post-operative Complications

#### Hematoma

Hematoma with ecchymosis around the involved knee occurred in 16 knees (3.9%) in ten patients in AMR group and 8 knees (8.1%) in five patients in AMR+PLR group. All subsided uneventfully within one month.

#### Persistent effusion

Nine knees (2.2%) in seven patients in the AMR group and 4 knees (4%) in three patients in the AMR+PLR group experienced persistent effusion for more than one month after this procedure. After initial treatment including resting, compression bandaging and anti-inflammatory medication, the effusion subsided spontaneously within 2 weeks in 8 knees. The other 5 knees in three patients required aspiration of the knee to relieve the discomfort.

#### Wound irritation

Irritable pain, catching or giving way sensation when moving the involved knees were commonly complained during the first post-operative month. This discomfort was tolerable in most patients after reassurance and would usually subside spontaneously. In 13 knees (2.6%) of 13 patients there was persistent discomfort due to wound irritation for more than three months. After conservative treatment including local heat, massage, and stretching exercise by bending knee passively, all alleviated within one year.

### Outcome

Of the 505 knees available for final review the subjective assessment was satisfactory in 432 (85.5%). Statistically it was less satisfactory in grade IV knees (59%) compared to other grades. In the AMR+PLR group, it was less satisfactory in grade III and IV knees compared to earlier grades (Table 2).

**Table 2:** Subjective outcomes of different grade of medial compartment OA stratified by type of surgery

**Grade**	**AMR (N=406)**					**AMR + PLR (N=99)**					**Total (%)**
	**E†**	**G**	**F**	**P**	**Sat. (%)**	**E**	**G**	**F**	**P**	**Sat. (%)**	
I	29 (78.4)	3 (8.1)	3 (8.1)	2 (5.4)	86.5	6 (50)	5 (41.7)	0 (0)	1 (8.3)	91.7	87.8
II	104 (63.8)	38 (23.3)	16 (9.8)	5 (3.1)	87.1	34 (72.4)	11 (23.4)	1 (2.1)	1 (2.1)	95.7	89.1
III	84 (49.4)	66 (38.8)	11 (6.5)	9 (5.3)	88.2	20 (54.1)	9 (24.3)	4 (10.8)	4 (10.8)	78.4*	86.5
IV	15 (41.7)	8 (22.2)	4 (11.1)	9 (25)	63.9*	0 (0)	0 (0)	1 (33.3)	2 (66.7)	0*	59.0*
Total	232 (57.1)	115 (28.3)	34 (8.4)	25 (6.2)	85.5	60 (60.6)	25 (25.2)	6 (6.1)	8 (8.1)	85.9	85.5

† *E* excellent, *G* good, *F* fair, *P* poor, *Sat*.: satisfied = E + G.

* Statistically significant by comparisons for each pair using paired *t* test (p<0.05).

For the 134 knees received in-depth outcome studies, the Knee Society pain scores statistically improved from 49.9 (SD 13.5) to 81.0 (SD 12.6) (p<0.001) in the AMR group and 53.0 (SD 11.0) to 76.7 (SD 16.1) (p<0.001) in the AMR+PLR group. The Knee Society function scores statistically improved from 53.5 (SD 15.3) to 71.1 (SD 14.4) (p<0.001) in the AMR group and 57.1 (SD 10.7) to 69.5 (SD 18.2) (p<0.001) in the AMR+PLR group (Table 3). All subscales of KOOS also improved statistically (Table 4). The FTA improved from 1.57 (SD 3.9) to 1.93 (SD 4.1) (p=0.03) and the JSW increased from 3.02 (SD 1.24) to 3.17 (SD 1.17) (p=0.01). The tilt angle of the patella decreased from 11.8 (SD 5.1) to 6.9 (SD 4.4) (p<0.0001) in the AMR+PLR group (N=29). The radiographic outcome revealed reversal of the degeneration process over the medial compartment in 82.1% of these knees (N=134) and 93.1% of the patellofemoral joint in the AMR+PLR group (N=29) (Table 5).

**Table 3:** Pre-operative and post-operative knee society score for different grade of medical compartment OA

	**AMR (N=105)**				**AMR + PLR (N=29)**			
**Grade**	**Pain (SD)**		**Function (SD)**		**Pain (SD)**		**Function (SD)**	
	**Pre-op.**	**Post-op.**	**Pre-op.**	**Post-op.**	**Pre-op.**	**Post-op.**	**Pre-op.**	**Post-op.**
I	37.7 (18.8)	92.3 (13.3)	48.3 (20.2)	78.3 (20.2)	-	-	-	-
II	53.0 (12.6)	84.1 (9.9)	54.0 (14.6)	73.2 (14.3)	53.6 (9.8)	80.7 (16.4)	56.6 (11.1)	71.6 (19.7)
III	47.8 (13.6)	76.4 (12.4)	53.7 (16.1)	68.7 (13.1)	52.8 (14.2)	69.8 (14.1)	57.8 (10.9)	65.0 (15.8)
IV	43.3 (12.8)	76.4 (20.6)	51.1 (16.9)	65.6 (18.1)	44.0 (N=1)	64.0 (N=1)	60.0 (N=1)	70.0 (N=1)
Total	49.9 (13.5)	81.0 (12.6)	53.5 (15.3)	71.1 (14.4)	53.0 (11.0)	76.7 (16.1)	57.1 (10.7)	69.5 (18.2)
P value	<0.001		<0.001		<0.001		<0.001	

**Table 4:** Pre-operative and post-operative KOOS of different grade of medial compartment OA

	**P**		**S**		**ADL**		**S/R**		**QOL**	
**Grade (N)**	**Pre-op. (SD)**	**Post-op. (SD)**	**Pre-op. (SD)**	**Post-op. (SD)**	**Pre-op. (SD)**	**Post-op. (SD)**	**Pre-op. (SD)**	**Post-op. (SD)**	**Pre-op. (SD)**	**Post-op. (SD)**
I (3)	68.3 (1.2)	69.3 (9.8)	73.0 (13.9)	61.7 (4.0)	58.3 (5.8)	74.7 (11.0)	58.7 (7.5)	53.3 (11.6)	55.3 (11.9)	73.0 (3.5)
II (75)	66.9 (18.9)	81.2 (14.9)	62.0 (20.0)	71.0 (15.9)	80.0 (16.5)	86.8 (13.3)	53.2 (31.5)	65.4 (25.1)	56.5 (22.7)	64.7 (18.8)
III (46)	52.8 (21.0)	75.2 (15.8)	50.0 (18.8)	64.7 (16.3)	65.7 (20.0)	80.1 (16.5)	32.2 (26.7)	49.8 (26.6)	42.4 (20.1)	57.1 (21.6)
IV (10)	49.0 (24.5)	74.6 (15.8)	49.6 (23.3)	65.4 (21.6)	69.7 (19.7)	77.9 (22.7)	33.5 (27.4)	55.5 (32.9)	35.7 (26.0)	55.1 (24.1)
Total (134)	60.8 (21.0)	78.4 (15.4)	57.2 (20.6)	68.2 (16.5)	73.9 (19.1)	83.5 (15.5)	44.6 (30.9)	59.0 (26.8)	50.1 (23.0)	61.6 (20.3)
P value	<0.001		<0.001		<0.001		<0.001		<0.001	

**Table 5:** Radiographic outcome of different grade of medial compartment OA stratified by type of surgery

	**Medial compartment (AMR and AMR+PLR, N=134)**				**PF compartment (AMR+PLR, N=29)**			
**Grade**	**Better**	**Same**	**Worse**	Rev. NC*	**Better**	**Same**	**Worse**	**Rev. NC**
I	0	3 (100)	0	3 (100)	0	0	0	0
II	26 (33.9)	38 (51.8)	11 (14.3)	64 (85.3)	19 (100)	0	0	19 (100)
III	24 (51.4)	11 (24.3)	11 (24.3)	35 (76.1)	7 (77.8)	0	2 (22.2)	7 (77.8)
IV	5 (62.5)	3 (22.2)	2 (25.0)	8 (80.0)	1 (100)	0	0	1 (100)
Total	55 (41.0)	55 (41.0)	24 (18.1)	110 (82.1)	27 (93.1)	0	2 (6.9)	27 (93.1)

* Reverse natural course = Better + Same.

### Evidence of Cartilage Regeneration

**Figure 2:**
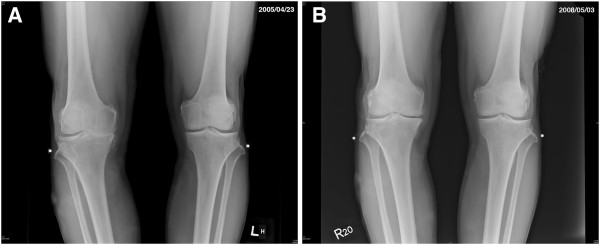
**An example of reversal of natural degenerative course by ACRFP.** A 70 years old male patient having grade IV OA over his right knee: **A**, pre-operative standing AP view showing grade IV OA over medial compartment of right knee; **B**, three years after ACRFP, the joint space reopened, the FTA improved from 7 degrees varus to 3 degrees varus.

**Figure 3:**
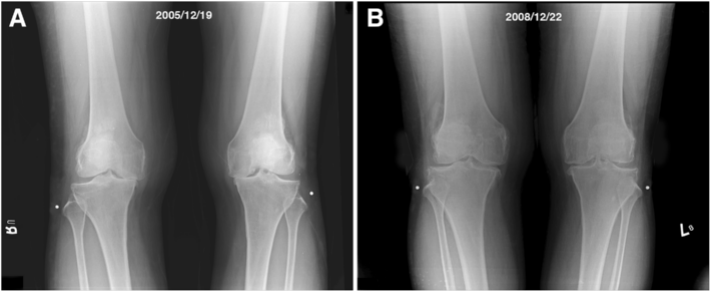
**Another example of reversal of natural course by ACRFP.A**, AP standing view of a 58 years old female patient having bilateral grade IV OA with subluxation of medial femoral condyle; **B**, three years after ACRFP, obvious improvement of the radiographic manifestation could be observed.

**Figure 4:**
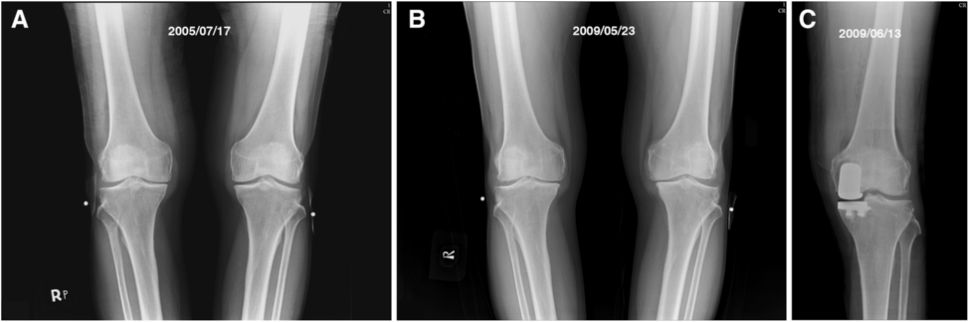
**An example of self-comparison showing the benefit of ACRFP.A**, pre-operative AP standing view of a 61 years old male with grade III OA over medial compartment of right knee and grade II OA over medial compartment of left knee. ACRFP was performed for his right knee; **B**, 46 months later, he came back due to marked disability over his left knee, the condition of his right knee was excellent. The degenerative process over the right knee is ceased compared to the progressively degenerated left knee; and, **C**, unicompartmental arthroplasty should be performed for his left knee.

**Figure 5:**
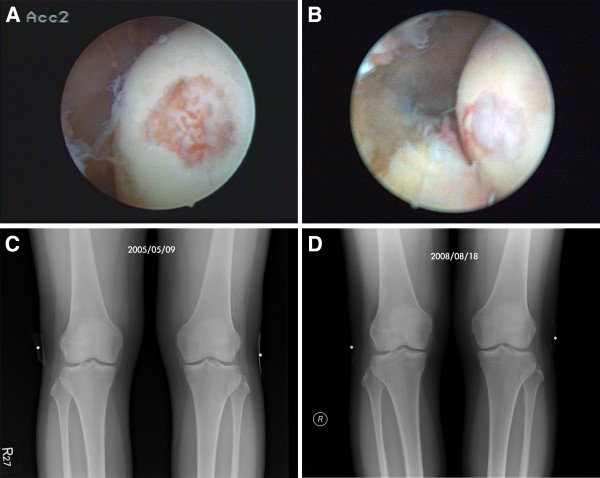
**Evidence of cartilage regeneration by second-look arthroscopy.** Cartilage regeneration demonstrated in the medial compartment of the left knee of a 56 years old lady having stage II OA: **A**, cartilage erosion was found over the medial femoral condyle opposite the removed pathologic medial plica; **B**, the defect was found regenerated during the second-look arthroscopy 3 years later when the same knee suffered from hemoarthrosis after a falling down accident; **C** and **D**, The grade II degenerative medial compartment did not show any progression.

In addition to the indirect evidence provided by radiographic images shown in Figures 2, 3, and 4, we had a chance to visualize the regeneration of a chondral defect when performing a second-look arthroscopy for a patient who suffered from hemoarthrosis due to an accident 3 years after the initial ACRFP for grade II osteoarthritis over the medial compartment of her right knee. The subjective outcome of her initial ACRFP was excellent (the Knee Society pain scores improved from 55 to 95, the Knee Society function scores improved from 35 to 80 and all subscales of KOOS also improved significantly: P- 36 to 94; S- 39 to 71; ADL- 53 to 97; S/R- 10 to 75; QOL- 6 to 88) and the degenerative medial compartment did not show any progression after the initial management according to her radiographic images (Figure 5, Additional file 2: Video 2).

## Discussion

In this report we present the clinical and radiographic outcomes of our procedure of arthroscopic cartilage regeneration facilitating procedure (ACRFP) for OA knees involving mainly medial and patellofemoral compartments. Overall, the subjective satisfactory rate was 85.5%. Even in grade IV OA, 59% of satisfactory rate could be anticipated after at least 3 years’ follow-up. Besides the improvement of clinical symptoms and life quality according to the KSS and KOOS surveys, revitalizing of the degenerated cartilage and reversal of the degeneration process were perceived in 81.2% of the knees by radiographic evaluation.

Therapeutic arthroscopic techniques including lavage, debridement, abrasional chondroplasty and microfracture are widely used for OA knee despite their unpredictable outcomes. A double-blinded, randomized, placebo-controlled trial to compare the effectiveness of arthroscopic lavage and arthroscopic debridement versus a sham procedure has been performed and the data suggest that the benefits of arthroscopy for the treatment of osteoarthritis of the knee are to provide subjective pain relief via a placebo effect [24]. The American Academy of Orthopaedic Surgeons (AAOS) clinical practice guideline on the treatment of OA knee also recommends against performing arthroscopy with debridement or lavage in patients with a primary diagnosis of symptomatic OA of the knee [25]. Dandy’s abrasion chondroplasty [26] was popularized in the late 1980s. In 1993, Bert et al. found it appears to offer little benefit over partial meniscectomy and debridement in the degenerative knee [8]. It was also thought that this technique provide unpredictable results. Concerns include the durability of the fibrocartilage repair tissue and thermal damage to subchondral bone and adjacent normal articular cartilage during this procedure [27]. Microfracture, a modification of chondroplasty, might provide increase in joint space by growth of fibrocartilage and achieve functional improvement for patients with full-thickness chondral defects in the osteoarthritic knees [28]. But an evidence-based systematic analysis declared that this technique could only provide short-term functional improvement. Shortcomings of this technique include limited hyaline repair tissue, variable repair cartilage volume and possible functional deterioration [12]. Autologous chondrocyte implantation (ACI) has become an accepted option for the treatment of chondral defects in carefully selected patients. Current recommendations limit this procedure to younger patients, as insufficient data are available to conclusively evaluate outcomes in patients older than 45 years, especially in OA patients [29]. The unconfirmed pathogenesis of OA knee and a lack of a clear consensus on the indications and surgical techniques as well as the variable outcomes make these commonly used arthroscopic techniques remain a source of controversy.

Abrasion or impingement phenomenon between the medial plica and the opposite medial femoral condyle has been described in recent studies in patients with medial compartment OA knee [30-33]. The consequent study [34] found that the repeated injuries elicited by this abrasion phenomenon might trigger IL-1ß production, thus enhance the expression of MMP-3. Their demonstration of the expression of IL1-ß mRNA, MMP-3 mRNA and MMP-3 in medial plica of early stage OA knee suggests that this structure and its interplay with the facing medial femoral condyle might play an important role in the pathogenesis of medial compartment OA knee. These findings coincide with the fact that eradication of this structure by arthroscopic release could be effective in symptom relief or even might modify the disease process of medial compartment OA knees [16,35].

Based on these constitutional studies, we proposed a concept that by a purposeful eradication of all prejudicial factors in the degenerative knee, the jeopardized cartilage would have the chance to regenerate by its innate healing response. In comparison with the uncertain beneficial mechanism and the diversity of outcomes of current popular arthroscopic techniques for osteoarthritis of the knee, our concept of ACRFP had more precise rationale of treatment. The main theme of ACRFP was to remove any abnormal abrasion or impingement phenomenon and reestablish soft tissue balance around the patellofemoral joint. For knees demonstrating medial abrasion phenomenon, medial release [16] was performed to relief the tension and abrasion between the tight, fibrotic and hypertrophied medial plica and the adjacent medial femoral condyle. On the other hand, in knees demonstrating lateral compression syndrome over patellofemoral joint, percutaneous lateral capsular release that has the benefits of tension release and denervation [36-40] was added. The immediate effect of ACRFP was obtained by releasing the tension around patella caused by chronically inflamed soft tissue and by eradication of the hypertrophied and inflamed synovium that may cause pain in these degenerative knees over the medial compartment and patellofemoral joint. Furthermore, by eliminating these stress/inflammatory responses that are the key events on the onset and progression of osteoarthritis, this procedure would also bring forth to long-term favorable effects as a consequence of the global improvement of the environment of the knee joint for cartilaginous regeneration.

Although articular hyaline cartilage was classically considered having no or low potential for regeneration [41-46], some still thought that it does have the capacity to grow and remodel extensively during pre- and post-natal development and after trauma [47,48]. Both direct and indirect evidence of articular cartilage regeneration have been reported after correction of varus deformity for osteoarthritis of the knee by some authors. According to their investigations about the beneficial effects of valgus osteotomy for medial gonarthrosis, the main repair feature was proliferation of fibrocartilage, which covered bone and areas of fibrillated cartilage and filled vertical clefts in hyaline cartilage. The hyaline cartilage was found to show an increased cellularity with numerous nests of proliferating chondrocytes [49]. There was also arthroscopic visible improvement of the articular surface after this procedure [50]. Moreover, it was observed by direct vision that mature regeneration was more frequently found in the knees with radiographic increased width of the medial joint space after high tibial valgus osteotomy, and this even happened in the knees with eburnation of the subchondral bone [51].

Recent studies also point out that, unlike the first impression of a more or less static tissue, articular cartilage shows a slow turnover. Anabolic and catabolic pathways were thought to be very much intermingled in articular cartilage metabolism [52]. Under normal conditions, chondrocytes maintain the dynamic equilibrium between synthesis and degradation of extracellular matrix components. In osteoarthritic states, however, there is a disruption of matrix equilibrium leading to progressive loss of cartilage tissue [53]. Thus, the aim of osteoarthritis therapy might neither to stimulate anabolism nor to knock down catabolism, but to titrate the balance of anabolic-catabolic activities. In this study, the clinical outcomes and the radiographic findings have given light to us that, by removal of all existed catabolic factors, the anabolism of the damaged cartilage might become dominant and regeneration unveiled.

Although it was claimed that compared with the extended weight-bearing anterior-posterior (AP) radiograph, posteroanterior (PA) imaging of the knee in 20–30 degrees flexion (the schuss position) increases the reproducibility of radiographic JSW measurements in OA knees [21], standing AP radiographic views of the knee have still been widely used to determine the rate of disease progression in OA knee by measuring joint space narrowing and bony alignment [21,54-59]. Contrary to our observation, they all obtained the common finding of progressive narrowing of the joint space as the natural course of OA knee. Never the less, in order to get more convincing evidence, we need more precise methodology such as magnetic resonance images study for the evaluation of cartilage regeneration. Properly conducted randomized control clinical trials are also warranted.

## Conclusions

According to the experience of performing this procedure, the data support our contention that arthroscopic cartilage regeneration facilitating procedure (ACRFP) is a good modality for the treatment of osteoarthritis of the knee joint if only medial and/or patellofemoral joint were involved. It could modify the natural course of this common disease. This novel concept of treatment might give hope to the majority of patients who are in the ambiguous stages of OA knee before arthroplasty could be beneficial for them.

## Competing interests

The authors declare that they have no competing interests.

## Authors’ contributions

SRL, CCH, and CWL were involved in the design of the study and performed the statistical analysis. SRL and CCH performed the operations and collected data. CWL evaluated the radiographic outcomes. SRL, CCH, and CWL were responsible for drafting the paper and revising it. And all authors commented on the draft. All authors have read and approved the final manuscript.

## Pre-publication history

The pre-publication history for this paper can be accessed here:

http://www.biomedcentral.com/1471-2474/13/226/prepub

## Supplementary Material

Additional file 1 **Video 1.** Arthroscopic medial release for medial abrasion phenomenon. An example of stage III medial compartment osteoarthritic knee with medial abrasion phenomenon treated by arthroscopic medial release is shown in this video clip. This is a 78 years old female suffering from left knee pain for more than 10 years. The arthroscopic examination revealed tightness of the medial facet of the patellofemoral joint and obliteration of the inferomedial region of the patella by hypertrophied medial plica and synovitis tissue. The abrasion phenomenon between fibrotic medial plica and medial femoal condyle could be reproduced by manipulation. After arthroscopic medial release, the medial gutter and the damaged medial femoral condyle could be clearly visualized and the medial abrasion phenomenon was also eliminated. Click here for file

Additional file 2 **Video 2.** Evidence of cartilage regeneration after ACRFP by a second-look arthroscopy. This 56 years old lady received ACRFP and have had experienced a satisfactory outcome until three years later when she suffered from a falling down accident with the same knee injured. We therefore had the chance to perform a second-look arthroscopy and visualized the evidence of cartilage regeneration over her previous chondral defect on the medial femoral condyle. Click here for file
